# Supplementary material for: CDCP1 Expression Is a Potential Biomarker of Poor Prognosis in Resected Stage I Non-Small-Cell Lung Cancer
Source: J Clin Med. 2022 Jan 11;11(2):341. doi: 10.3390/jcm11020341 (PMC8779436; doi:10.3390/jcm11020341)
Supplement: Supplementary file 1 [file jcm-11-00341-s001.zip › jcm-1521072-supplementary.pdf]

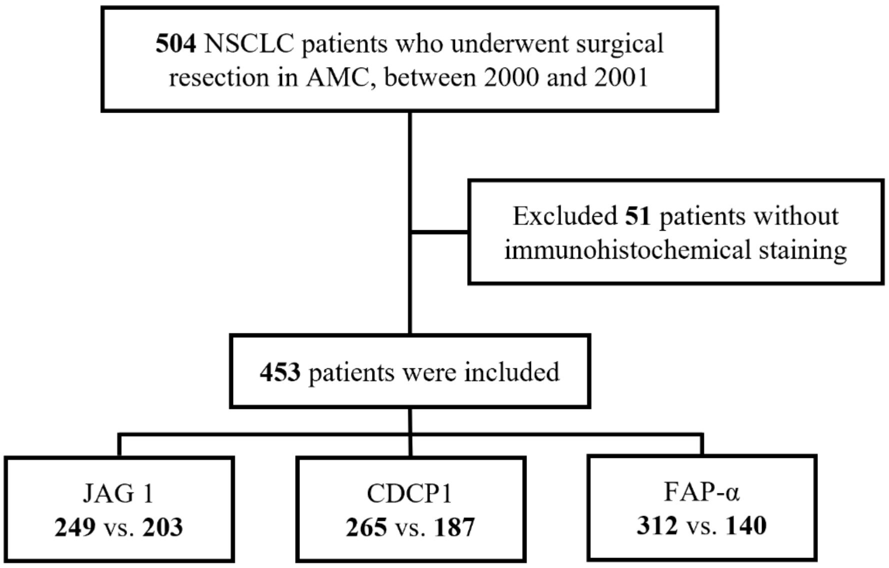

Figure S1. Flow sheet.

Table S1. Clinical characteristics of stage I patients. Median follow-up period: 96 months (IQR, 69–106).

	**All (*n* = 250)**	**Jagged-1 (+) (*n* = 143)**	***p* Value**	**CDCP-1 (+) (*n* = 125)**	**P value**	**FAP-α (+) (*n* = 178)**	***p* Value**
Age, median (range)	63 (56–70)	63 (56–70)		62 (55–70)		62 (57–70)	
≥65	108 (43.2)	60 (42.0)	0.595	50 (40.0)	0.381	77 (43.3)	0.977
Sex, male (%)	156 (62.4)	90 (62.9)	0.432	92 (73.6)	0.001	106 (59.6)	0.144
Ever-smokers, *n* (%)	145 (58.0)	83 (58.0)	0.499	86 (68.8)	0.002	97 (54.5)	0.077
Operation type			0.983		0.013		0.302
Lobectomy, *n* (%)	216 (86.4)	124 (86.7)		100 (80.0)		155 (87.1)	
Sublobar resection, *n* (%)	24 (9.6)	14 (9.8)		15 (12.0)		18 (10.1)	
Bilobectomy, *n* (%)	10 (4.0)	5 (3.5)		10 (8.0)		5 (2.8)	
Adjuvant Chemotherapy, *n* (%)	12 (4.8)	6 (4.2)	0.787	6 (4.8)	0.901	9 (5.1)	0.779
Histology			0.737		<0.001		<0.001
Squamous cell, *n* (%)	56 (22.4)	34 (23.8)		48 (38.4)		28 (15.7)	
Non-squamous, *n* (%)	191 (76.4)	109 (76.2)		77 (61.6)		150 (84.3)	
Five-year disease free survival	50 (20.0)	33 (23.1)	0.344	32 (25.6)	0.081	39 (21.9)	0.235
Duration, months, median (range)	23 (13–38)	27 (14–38)		18 (10–34)		24 (14–39)	
Five-year overall survival	45 (18.0)	28 (19.6)	0.691	30 (24.0)	0.045	31 (17.4)	0.705

Table S2. Risk factors for mortality in stage I patients assessed using Cox regression analysis.

	**Univariate analysis**		**Multivariate analysis**	
Parameter	HR (95% CI)	*p* Value	HR (95% CI)	*p* Value
Age (≥65)	3.296 (2.036–5.336)	<0.001	3.420 (2.082–5.619)	<0.001
Male	1.781 (1.067–2.972)	0.027	1.302 (0.728–2.327)	0.373
Ever-smokers	1.663 (1.024–2.702)	0.040	1.067 (0.422–2.699)	0.890
Operation type		0.037		0.531
Lobectomy	1		1	
Sublobar resection	2.224 (1.195–4.142)	0.012	1.230 (0.416–3.637)	0.708

Bilobectomy	1.485 (0.539–4.087)	0.445	1.448 (0.740–2.834)	0.279
Histology				
Adenocarcinoma	0.462 (0.289–0.738)	0.001	1.874 (0.253–13.875)	0.539
Squamous cell	2.219 (1.384–3.557)	0.001	1.907 (1.181–3.081)	0.008
Biomarker				
JAG1	1.132 (0.710–1.805)	0.604	1.228 (0.762–1.977)	0.398
CDCP1	1.595 (1.003–2.538)	0.049	1.530 (0.917–2.554)	0.104
FAP-α	1.064 (0.643–1.762)	0.808	1.103 (0.639–1.904)	0.725
Adjuvant Chemotherapy	0.243 (0.034–1.75)	0.160	0.239 (0.033–1.729)	0.156

Abbreviations; JAG1: Jagged-1, CDCP1: CUB-domain-containing protein 1, FAP- α: Fibroblast activation protein-alpha.
